# A comparison of tympanic and rectal temperatures in term NIGERIAN neonates

**DOI:** 10.1186/1471-2431-12-86

**Published:** 2012-06-25

**Authors:** Chika O Duru, Felix O Akinbami, Adebola E Orimadegun

**Affiliations:** 1 Department of Paediatrics and Child Health, Niger Delta University Teaching hospital, Bayelsa State, Nigeria; 2 Institute of Child Health, College of Medicine, University of Ibadan, Ibadan, Nigeria; 3 Department of Paediatrics, College of Medicine, University of Ibadan, Ibadan, Nigeria

**Keywords:** Rectal temperature, Tympanic temperature, Sensitivity, Specificity, Predictive values, Term neonates

## Abstract

**Background:**

Tympanic thermometry has come as a suitable alternative to traditional thermometry because of its safety and ease of use. However, it is still yet to gain wide acceptance in African settings due to conflicting results on its accuracy, thus rectal thermometry remains the gold standard in the newborn. The aim of this study was to compare tympanic and rectal temperatures in term Nigerian neonates.

**Methods:**

Rectal and tympanic temperatures were measured simultaneously in 300 consecutive term neonates between the ages of 37 and 42 weeks gestation using mercury-in-glass and the Infrared tympanic thermometers respectively. Paired *t* test, Pearson correlation coefficient and the Bland-Altman plot were used to compute data. Using rectal thermometry as gold standard, the sensitivity, specificity and predictive values of tympanic thermometry at various rectal temperature cut-offs were determined. Receiver Operating Curves (ROC) were constructed and the Areas Under the Curves (AUC) were compared.

**Results:**

The mean rectal temperature (37.34 ± 0.55°C) was significantly higher than the mean tympanic temperature (37.25 ± 0.56°C) (p < 0.001) with a mean difference of 0.09 °C ± 0.24 °C (95% CI: 0.06, 0.12). There was a strong positive correlation between the two measurements (r = 0.9; p < 0.001). Tympanic thermometry showed sensitivities ranging from 65% to 86% and specificities of 95% to 99% at rectal temperature cut-offs of 37.5°C to 38°C. The positive and negative predictive values of the tympanic temperatures at the various temperature cut-offs ranged from 82% to 93% and 80% to 98% respectively. Accuracy was noted to increase with higher temperatures as shown by the Receiver Operating Curves with the highest accuracy at the temperature cut-off of 38°C and AUC of 0.91.

**Conclusions:**

The sensitivity of tympanic thermometry was relatively low in detecting rectal temperatures despite the good correlation and agreement between them. The specificities and predictive values of tympanic temperatures in detecting rectal temperatures were high and accuracy increased with higher temperatures. Though using the tympanic route for measuring temperature in the newborn is relatively safe and non-invasive, its low sensitivity limits its use. Further studies would be required to further assess the accuracy of tympanic temperature measurements in the newborn.

## Background

The measurement of body temperature is an important parameter in neonatal care as it is useful in the assessment of clinical state and necessary for the provision of appropriate nursing support. Many routes have been used to record body temperature in the newborn; however a consensus on the best site has not been reached [1]. The rectal route has been adjudged the most accurate as it has been found to accurately represent core temperatures [1,2].

However, there are drawbacks to the use of the rectal route for temperature measurements in children. These include the risks of rectal perforation [3,4], transmission of infection [1,5,6] and mercury poisoning when mercury-in-glass thermometers are used [5,7,8]. Rectal temperature measurements have also been thought to lag behind core temperature measurements during periods of cold stress in shock states due to impaired perfusion to the rectum [1,2,5].

Tympanic thermometry, however, has come as a newer and safer alternative to the traditional temperature measurements. It is an excellent site for the measurement of core temperature as it is readily accessible and shares the same blood supply as the hypothalamus [1,9]. The infrared tympanic thermometers are non-invasive [10], provide rapid readings within a few seconds [9-11] and are not affected by the presence of cerumen or otitis media [5]. Previous studies done in different settings have shown that rectal temperatures compared with tympanic temperatures could have strong correlation [12-17] and mean differences ranged from 0.29 °C to 0.4 °C [13,16]. Despite the good correlation, there are still controversies on the accuracy of tympanic thermometry in the reports of studies in children; neonatal period [2,14,16,18,19] and beyond [10-15,17]. This study aims to compare temperatures taken through the rectal and tympanic routes in term neonates to determine whether tympanic thermometry can accurately replace rectal temperature measurements as a sensitive and specific route of temperature measurement.

## Methods

This prospective cross sectional study was carried out at the University College Hospital, Ibadan, Nigeria over a 6 month period. Three hundred neonates, whose weights were appropriate for gestational age (37 and 42 weeks) admitted on the neonatal wards, lying in wards and emergency wards of the hospital were studied. Informed consent was obtained from the parents of the babies. Ethical approval was obtained from UI/UCH Ethics Committee (Approval number: UI/EC/09/0122). The children were seen between the hours of 8.00 am and 6.00 pm during periods of normal vital sign monitoring. Ambient temperatures throughout the study period ranged between 25 °C and 34 °C.

Conventional mercury-in-glass thermometers were used for rectal temperature measurements while the Infrared tympanic thermometer (BRAUN® Thermo scan ear thermometer model IRT 4520) was used for the tympanic temperature measurements. The neonates’ body temperatures were taken simultaneously from the two sites by two independent observers. The principal investigator (corresponding author) took the tympanic temperature measurements while the assistant (nurse) who had been well trained, inserted the mercury-in-glass thermometers for rectal temperature measurement at the same time.

In measuring temperatures, the anal openings of the babies were cleaned and a mercury-in-glass thermometer was lubricated with Vaseline and inserted within a depth of 2-3 cm into the rectum. The rectal thermometer was left for 3 minutes in the rectum to ensure stabilization before being read by the nurse. All the mercury-in-glass thermometers were shaken to decrease the reading to below 35°C before each temperature measurement. Simultaneously, the Infrared tympanic thermometer with a probe cover was placed into the right external auditory canal after an ear tug to straighten the canal and directed towards the eye to a depth of 0.5 to 1 cm. The probe was held in the same position until a single beep was heard (usually about 2 seconds) signifying the end of the temperature measurement which was then read by the principal observer. The probe cover was discarded and a new one placed on the probe for the next measurement. After the tympanic temperature measurements, the ear was examined with an auroscope to rule out the presence of debris, meconium or pus. No case of otitis media was recorded in the 300 neonates recruited for the study.

All the collected data were analyzed by an author who was blinded to the procedure. Statistical analysis was done using the Statistical Package for Social Sciences (SPSS) for Windows (Inc. Chicago USA, 2001). Paired *t* test was used to compare the mean temperature readings from the two routes. Correlation was determined by the Pearson correlation coefficient while the extent of agreement was assessed with the Bland-Altman plot. Using various rectal temperature cut-offs as gold standard from 37.5 °C and at 0.1 °C differences above that till 38 °C, the sensitivities, specificities, positive and negative predictive values of tympanic temperatures were determined. Receiver Operating Curves (ROC) were drawn for the two temperature measurements and the Areas Under the Curve were compared. The level of significance was taken to be p < 0.05.

## Results

**Figure 1:**
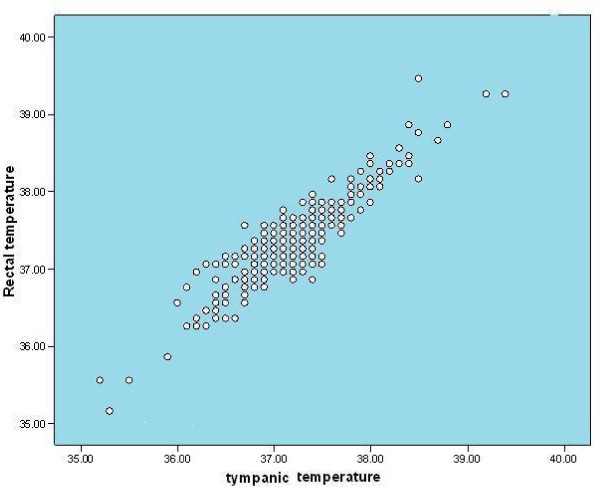
** The relationship between rectal temperatures and tympanic temperatures.** There is a strong positive correlation between rectal and tympanic temperatures with high rectal temperatures associated with high tympanic temperatures.

The mean gestational age of the neonates who participated in this study was 38.9 ± 1.6 weeks. Of the 300 neonates, 166 (55.3%) were male while 134(44.7%) were female with a male/female ratio of 1.2:1. The mean age at the time of recruitment was 6.63 ± 6.98 days. Both rectal and tympanic temperatures ranged from 35 °C to 39.4 °C. The mean rectal temperature (37.34 ±0.55 °C) was significantly higher than the mean tympanic temperature (37.25 ±0.56 °C;p < 0.001). There was a significant positive correlation between rectal and tympanic temperatures (r = 0.91; p < 0.001).As shown in Figure 1, high rectal temperatures were associated with high tympanic temperatures.

**Figure 2:**
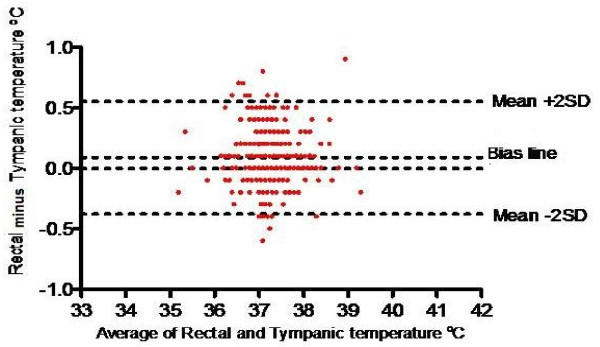
** Bland Altman Plot of the agreement between mean rectal –tympanic temperatures and differences between the temperature measurements.** It shows that most of the data points are tightly clustered around the zero line of the difference between the two temperature readings with only 8.3% of the readings falling outside the 95% level of confidence.

The Bland-Altman plot (Figure 2) showed that most of the data points were tightly clustered around the zero line of the difference between the two temperature readings with only 8.3% of the readings falling outside the 95% level of confidence. The average difference between the mean of both rectal and tympanic temperatures was 0.09 ± 0.24 °C (95% CI: 0.06, 0.12).

**Figure 3:**
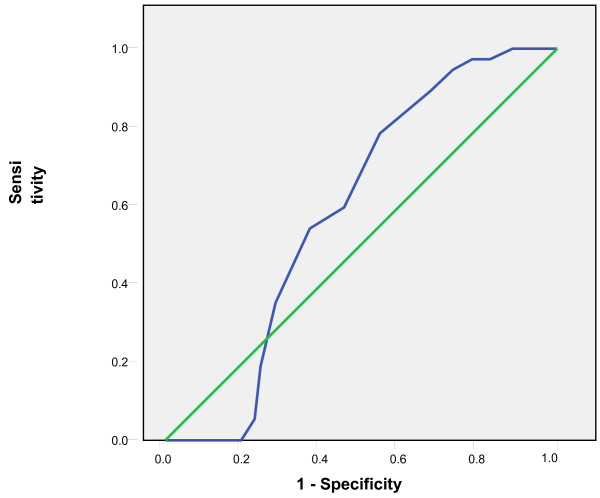
**Receiver Operating Curve for the accuracy of tympanic temperature measurements versus rectal measurements at temperature cut-off of 37.5°C.** The Area Under the Curve is 0.6 (95%CI: 0.5, 0.7)

**Figure 4:**
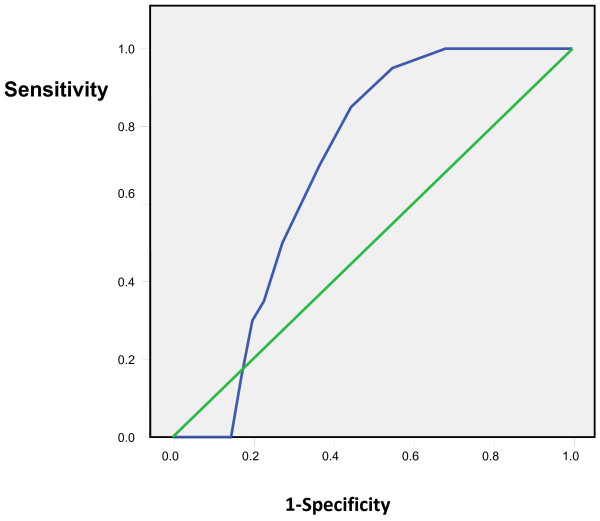
**Receiver Operating Curve for the accuracy of tympanic temperature measurements versus rectal measurements at temperature cut-off of 37.6°C.** The Area Under the Curve is 0.7 (95%CI: 0.6, 0.8).

**Figure 5:**
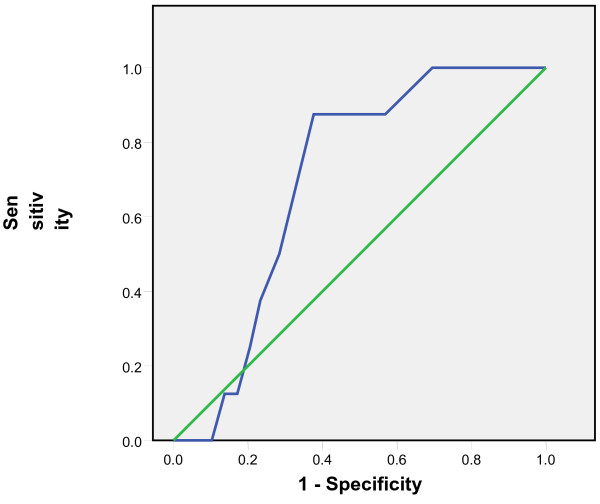
**Receiver Operating Curve for the accuracy of tympanic temperature measurements versus rectal measurements at temperature cut-off of 37.7°C.** The Area Under the Curve is 0.7 (95% CI: 0.6, 0.8).

**Figure 6:**
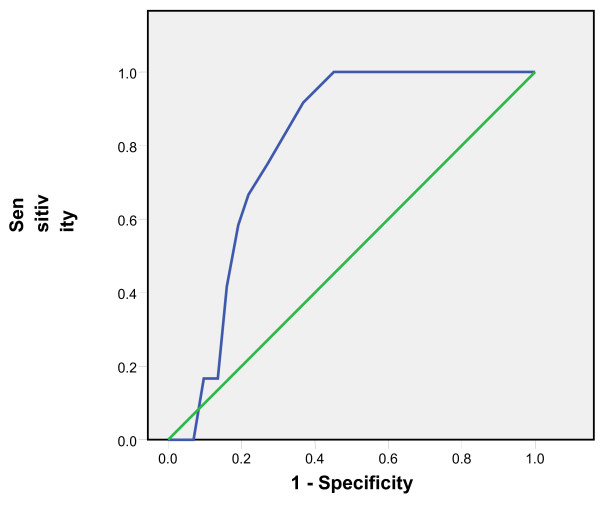
**Receiver Operating Curve for the accuracy of tympanic temperature measurements versus rectal measurements at temperature cut-off of 37.8°C.** The Area Under the Curve is 0.8 (95% CI: 0.7, 0.9).

**Figure 7:**
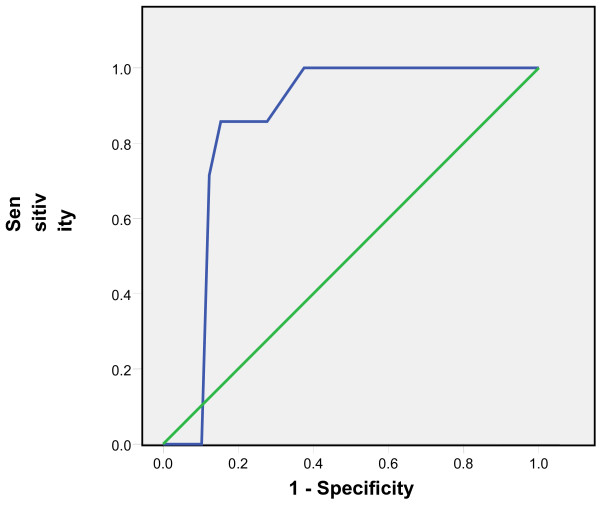
**Receiver Operating Curve for the accuracy of tympanic temperature measurements versus rectal measurements at temperature cut-off of 37.9°C.** The Area Under the Curve is 0.85(95%CI: 0.8, 0.9).

**Figure 8:**
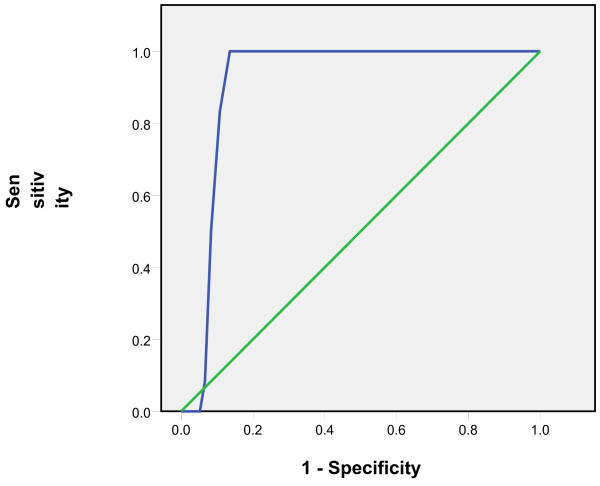
**Receiver Operating Curve for the accuracy of tympanic temperature measurements versus rectal measurements at temperature cut-off of 38°C.** The curve lying closest to the y axis is the most accurate and this is shown in this ROC at temperature cut-off of 38 °C and Area Under the Curve of 0.91 (95% CI: 0.89, 0.94).

Tympanic temperatures ≥37.5 °C and subsequent 0.1 °C differences up to 38 °C were considered as rectal temperatures cut-offs and the comparison with tympanic measurements showed sensitivities of 65% to 86% and specificities of 95% to 99%. The positive predictive values of the tympanic measurements ranged from 82% to 93% while the negative predictive values ranged from 80% to 98% (Table 1). The Receiver Operating Curves (ROC) showed the highest accuracy at the temperature cut-off of 38°C and Area Under the Curve of 0.91(Figures 3, 4, 5, 6, 7, 8).

**Table 1:** Comparison of the sensitivity, specificity, positive and negative predictive values of tympanic temperatures at rectal temperature cut-offs

**Parameters (%)**	**Temperature cut-offs**					
	**≥ 37.5 °C**	***≥*****37.6 °C**	***≥*****37.7 °C**	***≥*****37.8 °C**	***≥*****37.9 °C**	***≥*****38 °C**
Sensitivity	65	73	80	84	86	76
Specificity	96	95	95	98	99	99
Positive predictive values	91	86	82	92	93	93
Negative predictive values	80	90	95	96	98	97

## Discussion

Monitoring the body temperature of neonates is a clinically necessary procedure to assess the overall patients’ status. There is a need to accurately measure temperature as any alteration could signal the onset of infection, serious reactions to medications, critical loss of heat from exposure and other specific disease conditions. Temperatures get measured at the least as frequently as every three hours in a typical neonatal care unit. Thus, the importance of painless, accurate and rapid measurement of temperature cannot be over-emphasized. Temperature measurements through the rectal route have been considered the gold standard method in children but there is evidence that it is associated with many risks and complications [1-8]. On the other hand, measurement through the tympanic route using the infrared tympanic thermometer has been adjudged to be easier and quicker for assessment of body temperatures [1,9].

In this study, we found a strong positive correlation between rectal and tympanic temperatures as earlier reported in other studies in Nigeria [12,17]. The mean rectal temperature taken with the mercury-in-glass thermometer was significantly higher than the mean tympanic temperature taken with the Infrared tympanic thermometer by 0.09 °C. This agrees with findings reported by Edelu *et al.*[17] who also used the mercury-in-glass thermometer to measure rectal temperatures. Uslu *et al.*[19] in their study also reported similar findings even though sick newborns were studied. The reasons postulated for the higher rectal temperatures noted was that the rectum was better insulated than the tympanic membrane from external temperature changes [14].

This difference (0.09°C), though apparently small, is significant when measuring temperature in the neonate where accuracy is crucial for important management decisions. Akinyinka *et al.*[12] also reported a comparable mean difference between rectal and tympanic temperatures of 0.08 °C but the difference was not statistically significant. The lack of statistical significance in the mean temperature difference in the study by Akinyinka *et al.*[12] could be attributed to the smaller sample size compared to the present study. In an earlier study, Weiss *et al.*[16] got a difference of 0.4 °C between both rectal and tympanic measurements but the electronic thermometer was used to measure rectal temperatures instead of mercury-in-glass thermometer used in our study.

In the present study, we used various rectal temperature cut-offs at 0.1 °C intervals between 37.5 °C and 38 °C to compute the sensitivities, specificities and predictive values of tympanic temperature measurements. It was noted that the sensitivities of tympanic temperatures in detecting rectal temperatures at cut-offs greater than or equal to 37.5 °C were low but increased with higher rectal temperatures (65% -86%). The temperature measurements from the two sites however showed very high specificities ranging from 95% to 99%. These findings suggest that tympanic thermometry for detection of fever in the neonates may not actually be good for screening purposes but has improved diagnostic accuracy as body temperature increases. The increasing sensitivities at higher temperature values was also observed by Edelu *et al.*[17]. Moreover, the fact that our data showed relatively low sensitivities at lower temperature cut-offs implies that at these temperature cut-offs, about 14-35% of neonates found to be ‘febrile’ as defined by any of these rectal cut-offs might be missed if the tympanic thermometer was used. The high specificities however indicated that a neonate adjudged to be hypothermic or pyretic using the rectal route will also be detected as hypothermic or pyretic by the tympanic route.

The positive and negative predictive values were also high, ranging from 82% to 93% and 80% to 98% respectively and these values increased with increasing temperatures cut-offs. The Receiver Operating Curves also supported these findings with greater accuracy at higher temperatures than lower ones. This implies that at higher temperature cut-offs, tympanic temperatures are more sensitive and specific and accurate in determining rectal temperatures than at relatively lower ones.

A limitation of this study was that we did not have a true measure of core body temperature to compare our temperature findings with. Core temperature refers to the measurement that most closely reflects the temperature in the blood flowing through the branches of the carotid arteries to the hypothalamus [5]. However, since the hypothalamus is inaccessible, other sites of core temperature measurement include the pulmonary artery, oesophagus, nasopharynx and bladder [1]. Since these sites are not routinely used in everyday practice and the patients in our study were apparently healthy neonates, the rectum was used as it has been shown to accurately approximate core temperature [1].

## Conclusions

It is concluded that though using the tympanic route for measuring temperature in the neonate is relatively safe and non invasive, its low sensitivity limits its use despite the good correlation and agreement between the measurements taken from the two routes. Further studies will be needed to further evaluate its utility in neonates especially in resource poor countries.

## Competing interests

The authors declare that they have no competing interests.

## Authors’ contributions

COD conceived the study, participated in its design, coordination and acquisition of data and drafting of the manuscript. FOA also participated in conception of the study, supervised its conduct and drafting of the manuscript and revised it. AED carried out the data analysis, participated in interpretation of data and drafting of the manuscript. All authors read and gave the final approval of the version to be published.

## Pre-publication history

The pre-publication history for this paper can be accessed here:

http://www.biomedcentral.com/1471-2431/12/86/prepub
